# Epidemiology, use, and practice of the intraosseous route in an out-of-hospital emergency department: a retrospective cross-sectional study

**DOI:** 10.3389/fpubh.2024.1375431

**Published:** 2024-04-17

**Authors:** Amaya Burgos-Esteban, Manuel Quintana-Diaz, Valvanera Cordón-Hurtado, Marta Giménez-Luzuriaga, Iván Santolalla-Arnedo, Regina Ruiz de Viñaspre-Hernández, Vicente Gea-Caballero, Jose Angel Santos-Sánchez, Noelia Navas-Echazarreta, Antonio Rodríguez-Calvo, Pilar Sánchez-Conde, Raúl Juárez-Vela

**Affiliations:** ^1^Doctoral Program in Medicine and Surgery, Autonomous University of Madrid, Madrid, Spain; ^2^Department of Nursing, Research Group in Care, University of La Rioja, Logroño, Spain; ^3^Rioja Health Service, Logroño, Spain; ^4^Intensive Care Department, University Hospital La Paz, Madrid, Spain; ^5^Faculty of Health Sciences, International University of Valencia, Valencia, Spain; ^6^Faculty of Medicine, University Hospital of Salamanca, Salamanca, Spain

**Keywords:** vascular access devices, emergencies, emergency medical services, out-of-hospital cardiac arrest, emergency care

## Abstract

**Introduction:**

The Spanish Emergency Medical Services, according to the model we know today, were formed during the 80s and 90s of the 20th century. The Health Emergency Service (EMS), 061 La Rioja, began to assist the population of La Rioja in November 1999. An essential part of the mission of the SES is the provision of care and the transfer of critical patients using advanced life support unit (ALSU) techniques. In daily practice, out-of-hospital emergency services are faced with situations in which they must deal with the care of serious or critically ill patients, in which the possibility of being able to channel peripheral vascular access as part of ALSU quickly may be difficult or impossible. In these cases, cannulation of intraosseous (IO) vascular access may be the key to early and adequate care.

**Aim:**

This study aimed to determine the incidence and epidemiology use of IO vascular access in SES 061 La Rioja during the year 2022.

**Matherial and methods:**

We performed observational retrospective cross-sectional studies conducted in 2022. It included a population of 4.364 possible patients as a total of interventions in the community of La Rioja in that year.

**Results:**

A total of 0.66% of patients showed a clinical situation that required the establishment of IO vascular access to enable out-of-hospital stabilization; this objective was achieved in 41.3%. A total of 26.1% of patients who presented with cardiorespiratory arrest (CA) were stabilized, while 100% presented with shock and severe trauma.

**Discussion:**

IO vascular access provides a suitable route for out-of-hospital stabilization of critically ill patients when peripheral vascular access is difficult or impossible.

## 1 Introduction

The Spanish Emergency Medical Services (EMSs), according to the model we know today, were formed during the 80s and 90s of the 20th century (1). The EMS, 061 La Rioja, began to assist the population of La Rioja in November 1999 (2). An essential part of the mission of the ES is the provision of care and the transfer of critical patients using advanced life support unit (ALSU) techniques, as described in Royal Decree 1030/2006, of 15 September, which establishes the portfolio of common services of the National Health System and the procedure for updating it (3–5). The Real Spanish Academy of Language (RAE) defines the word critical as “very difficult or very serious,” including the terms crucial, decisive, delicate, and serious as synonyms. The RAE defines an emergency as “a situation of danger or disaster requiring immediate action” (6). During the year 2022, emergency services in Spain attended a total of 1,344,085 calls that required the displacement of an ALSU vehicle, either by land (type C vehicle, 1,325,238) or air equipment (HEMS, 18,847), for their resolution. In the Autonomous Community of La Rioja, a total of 4,364 patients were attended by type C, ALSU ambulance vehicles (7).

In daily practice, out-of-hospital emergency services are faced with situations in which they must deal with the care of serious or critically ill patients, in which the possibility of being able to channel peripheral vascular access as part of ALSU quickly may be difficult or impossible. In these cases, cannulation of intraosseous (IO) vascular access may be the key to early and adequate care (8, 9). In the autonomous community of La Rioja, the automatic intraosseous access (IOA) device has been available since January 2020, which is suitable for the establishment of IO vascular access in both adults and children in emergencies as an alternative to peripheral vascular access (10).

Therefore, this study aimed to determine the incidence and epidemiology use of IO vascular access in SES 061 La Rioja during the year 2022.

## 2 Methods

An observational retrospective cross-sectional study was conducted in 2022. During that year, a total of 4,364 possible patients were attended to in emergencies. Our inclusion criteria were all patients who had undergone IOA by the ALSU Service during working hours.

**Figure 1:**
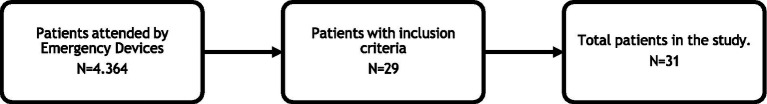
Flow chart.

Figure 1 shows the flow chart. Briefly, 4.364 attended in the Autonomous Community of La Rioja. A total of 29 subjects were recruited; however, 2 were added to present more than one intraosseous vascular access. We excluded all patients who did not have intraosseous access or was attended for other medical devices outside of La Rioja.

The study protocol was designed by the Declaration of Helsinki, and ethical approval was granted from the Ethics Committee, verification code nOjsSvXLLNRvnfVfPr299EBR2092SU2L.^1^

### 2.1 Study variable and definitions

The necessary data were collected from the nursing professionals who performed the IO cannulation technique under study. La Rioja covers a population of 319,485 people. To provide its service during the year 2022, it had an emergency coordination center and three ALSUs located in the towns of Logroño, Calahorra, and Haro, each of these units has an IOA. This device consists of a motor and needles of three sizes: pink (1.5 cm), blue (2.5 cm), and yellow (4.5 cm), all with a 15G gage. All the nursing and medical staff of the service participated in the study, proceeding to the recording of the data variables when the inclusion criteria were met.

For the coding of the clinical and epidemiological data, a form established for this purpose was used and communicated to the principal investigator of the study. All the staff of the Emergency System of La Rioja were informed about the purpose of this research and the need to communicate with the principal investigator if an IOA was used. A double-coding system was used to guarantee the privacy of the data. The variables studied were the resource in charge of care; sex, age, and weight of the patient; clinical situation, justification for the choice of IO vascular access, selected needle location and size; drugs and fluids infused; and complications encountered during placement, use, and removal of the needle.

### 2.2 Statistical analysis

Statistical analysis was performed using IBM SPSS version 27.0 (IBM Corporation, Armonk, NY, USA) Categorical variables are reported as frequencies (%), and continuous variables are reported as averages ± standard deviations. For categorical variables, the χ^2^ or Fisher’s exact test was used, or the Mann–Whitney U-test (if the distribution was non-normal) was applied to compare continuous variables. A normal distribution was revealed when a *p*-value of >0.05 was found by the Kolmogorov–Smirnov test. Pearson’s correlation test was used to investigate the relationship between clinical and epidemiological variables. On the other hand, univariate and multivariate logistic regression analyses were conducted to investigate the possible association between IOA and complications.

## 3 Results

The data collected in the study show that a total of 4,364 patients were attended by the ALSU of the ES 061 La Rioja; 29 required IOA for their clinical approach, 20 men and 9 women. Of the total number of patients, four were children, and 100% were under 10 years of age (Appendix I: table of age and sex of patients). In 29 cases, a single IOA was established, with a second access being necessary in the case of two patients, one male and one female, making a total of 31 cannulations performed. The male patient was in shock and progressed to cardiorespiratory arrest (CA). The first access in the humeral location did not perfuse adequately, so a second cannulation was performed in the proximal tibial location, achieving perfect functioning, which allowed stabilization and transfer of the patient to the reference hospital.

The woman was in CA; IO vascular access was established in the tibia after two unsuccessful attempts at peripheral venous cannulation. After verifying that it did not work properly, as it required high external pressure for infusion, a new IO vascular access was established in the proximal tibia (contralateral), which worked properly. Subsequently, this second access was extravasated. In both cases, out-of-hospital stabilization and transfer to the hospital were achieved.

Sixty-nine percent of the patients were attended by ALSU 1 (City of Logroño), 17.2% by ALSU 2 (City of Calahorra), and 13.8% by ALSU 3 (City of Haro). There were statistically significant differences in terms of the resources used for the clinical situation presented by the patient (*p*-value = 0.005). A total of 82.6% of cases of CA were treated in the city of Logroño (ALSU 1), compared to 13% in the city of Calahorra (ALSU 2) and 4.3% in the city of Haro (ALSU 3). Of the cases of shock, 50% occurred in Haro (ALSU 3). Approximately half of the severe trauma occurred in Calahorra (ALSU 2) and half in Haro (ALSU 3). The relationship between the clinical situation and the resource providing care is summarized in Table 1.

**Table 1:** Relationship between clinical situation-assigned resource.

Clinical situation	ALSU* 1		ALSU* 2		ALSU* 3		Fisher’s exact test
	*N*	%	*N*	%	*N*	%	
Cardiorespiratory arrest	19	82.6	3	13.0	1	4.3	*p*-value = 0.005
Shock	1	25.0	1	25.0	2	50.0	
Severe trauma	0	0.0	1	50.0	1	50.0	

*ALSU, advanced life support ambulance.

The administration of the prescribed therapy through IO vascular access achieved out-of-hospital stabilization in 41.4% of patients, compared to 58.6% of patients who died *in situ* (Appendix II: List of drugs and fluids administered). There is a statistically significant difference regarding the relationship between the clinical situation of the patient and out-of-hospital stabilization (*p*-value = 0.002). Of the cases of CA, 73.9% died out-of-hospital, compared to 100% of cases of shock and severe trauma that were stabilized out-of-hospital. About the resource that provided care to the patient, 35% of patients in ALSU 1, 40% in ALSU 2, and 75% in ALSU 3 were stabilized, although no statistically significant differences were found (Fisher’s exact test, *p*-value = 0.369) (Table 2).

**Table 2:** Relationship between out-of-hospital stabilization and clinical situation and assigned resources.

Clinical situation	Death *in situ*		Out-of-hospital stabilization		Total	Fisher’s exact test
	*N*	%	*N*	%	*N*	
Cardiorespiratory arrest	17	73.9	6	26.1	23	*p*-value = 0.002
Shock	0	0	4	100	2	
Severe Trauma	0	0	2	100	4	
Unit of emergency						
*ALSU 1	13	65.0	7	35%	20	*p*-value = 0.369
*ALSU 2	3	60.0	2	40%	5	
*ALSU 3	1	25.0	3	75%	4	

*ALSU, advanced life support ambulance.

The use of continuous perfusion according to the clinical situation showed statistically significant differences with a *p*-value of 0.017. It was used in 100% of patients with severe trauma, in 50% of patients in shock, and in 17.4% of patients with *CA.* Table 3 shows the relationship between continuous fluid perfusion and the absence of fluid perfusion.

**Table 3:** Relationship between clinical situation - continuous fluid perfusion.

Clinical situation	No continuous infusion		Continuous infusion		Fisher’s exact test
	*N*	%	*N*	%	
Cardiorespiratory arrest	19	82.6	4	17.4	*p*-value = 0.017
Shock	2	50.0	2	50.0	
Severe trauma	0	0.0	2	100.0	

The decision to cannulate IOA was taken in 6.9% of the cases after one failed attempt at peripheral venous cannulation, 20.7% after two failed attempts, and 3.4% after more than two failed attempts, with cannulation being justified by the clinical situation of the patient in 69% of the cases. There were significant differences in the justification for using IOA according to the clinical situation of the patient (Fisher’s exact test, *p*-value = 0.003). In CA, the nurse’s criterion prevailed in 82.6%; in shock, it was cannulated after two unsuccessful attempts in half of the patients; and in severe trauma, it was cannulated after two unsuccessful attempts in all cases. Table 4 summarizes the relationship between the clinical situation and the justification for the choice of access.

**Table 4:** Relationship between the justification for the establishment of intraosseous vascular access and clinical situation.

Clinical situation	Cardiorespiratory arrest		Shock		Severe trauma		Test de Fisher
Justification for decision	*N*		%		*N*		%
One failed attempt	2	8.7	0	0	0	0	*p*-valor = 0.003
Two failed attempts	2	8.7	2	50.0	2	100	
>2 attempts	0	0	1	25.0	0	0	
Clinical situation	19	82.6	1	25.0	0	0	

45.2% of the accesses were placed in the humeral head, 51.6% in the proximal tibia, and 3.2% in the distal femur, and no access was cannulated in the distal tibia. In 48.4% of the cases, a 45 mm needle was used, 51.6% used a 25 mm needle, and the 15 mm needle was not used on any occasion. Table 5 summarizes the information on the access place.

**Table 5:** Access place.

Accesses place		
	N	%
Humeral head	14	45.2
Proximal tibia	16	51.6
Distal tibia	0	0.0
Distal femur	1	3.2

There were significant differences in the type of needle used according to the vital stage of the patient. For children, 25 mm needles were used in 100% of the cases, while for adults, the 25 mm needle was used in 40% and the 45 mm needle in 60%. There were no significant differences in the location of the access according to life stage (*p*-value = 0.222). Table 6 summarizes the information on the life stage, locations, and needle size.

**Table 6:** Relationship between life stage-needle location and size.

Vital Stage	Adult		Childhood		Total	Fisher’s exact test
Localization	*N*		%		*N*	
Humeral head	13	52.0	1	25.0	14	*p*-value = 0.222
Proximal tibia	12	48.0	2	50.0	14	
Distal tibia	0	0.0	0	0.0	0	
Distal femur	0	0.0	1	25.0	1	
Needle size						
15 mm	0	0.0	0	0.0		*p*-value = 0.041
25 mm	10	40.0	4	100	14	
45 mm	15	60.0	0	0.0	15	

No professional recorded any complications related to needle insertion. External pressure was necessary to achieve an adequate infusion rate on 9.7% of occasions. Significant differences were found in the need for external pressure depending on the clinical situation of the patient, with 50% requiring external pressure in both severe trauma and shock. Table 7 summarizes the data regarding this relationship.

**Table 7:** Relationship between clinical situation-need for external pressure.

	Need for external pressure				
	Not		Yes		Test de Fisher
Clinical situation	N		%		
Cardiorespiratory arrest	23	100.0	0	0.0	*p*-value = 0.005
Shock	2	50.0	2	50.0	
Severe trauma	1	50.0	1	50.0	

## 4 Discussion

This study aimed to determine the incidence and epidemiology of IO vascular access use in ALSU 061 La Rioja during the year 2022. The Autonomous Community of La Rioja had a population of 319,892 inhabitants in 2022, according to the Statistical Institute of La Rioja. The distribution of the population served by different resources was as follows: 69% in the city of Logroño, 17.2% in the city of Calahorra, and 13.8% in the city of Haro, corresponding to reference populations of 199,000, 75,000, and 45,000 inhabitants, respectively. The majority of care in the Middle Rioja Region (82.6% of the Autonomous Community) was provided due to the location of the capital, Logroño, which had a population of 150,020 inhabitants in 2022 (11).

Of the 4,364 cases provided by ALSU during the year 2022, 29 patients (0.66%) presented clinical situations that required the establishment of IO vascular access to enable out-of-hospital stabilization and transport of the patient to the hospital. This objective was achieved in 12 out of the 29 cases (41.3%). It is important to note that this objective was successfully met in 100% of patients who presented with shock and severe trauma, demonstrating that IO vascular access is a suitable method for administering fluids, either continuously or intermittently, as well as medications. This approach is effective for stabilizing critical patients when cannulating a peripheral venous line is either difficult or impossible (12–15). Furthermore, the out-of-hospital stabilization of 26.1% of patients with cardiac arrest (CA) who received IO vascular access and 100% of patients with severe trauma and shock indicate that the early implementation of IO vascular access increases the chances of out-of-hospital stabilization for critically ill patients in whom peripheral vascular access is difficult or impossible (8, 9, 16).

Regarding the early establishment of IO vascular access, deciding to cannulate based solely on the clinical situation of the patient was easier in cases of patients experiencing CA, which accounted for 82.6% of the cases. In these cases, IOA was chosen as the first option in 25% of shock cases and was not used in any cases of severe trauma. It is important to highlight that nursing professionals attempted peripheral venous cannulation one or more times before opting for IOA in these clinical scenarios. Following the study and confirmation that IOA is effective for stabilizing patients in shock and severe trauma, it is advisable to instruct nursing professionals to prioritize early cannulation of IO vascular access. This approach can help prevent the progression of the condition to a more challenging out-of-hospital stabilization scenario (10, 13, 17).

Regarding the most appropriate puncture location, the study aligns with the current trend favoring insertion in the humeral head. This is supported by the fact that 66.6% of stabilized patients had access to the humeral head, while 33.3% had access to the proximal tibia. An equal number of patients received humeral and tibial accesses when considering the total number of patients (8, 10).

It is worth noting that the professionals did not encounter any difficulties in establishing IOA in any of the locations studied. This success can be attributed to the adequate training of the nursing staff in understanding the available devices, insertion techniques, and necessary nursing care to perform the procedure (13).

A significant body of evidence suggests that the IO route of infusion is pharmacokinetically equivalent to the intravenous (IV) route and superior to the intramuscular and endotracheal routes for administering drugs in animal models. Additionally, indications for IO access are linked to explosive attacks and mass shootings, indicating that expanded utilization of the IO route and military resuscitation strategies could be beneficial. The IO route is also valuable for fluid resuscitation in the management of diarrheal and hemorrhagic infectious disease outbreaks, providing a rapid and safe vascular access option (18, 19). In line with other studies comparing IO access and peripheral IV access, our research demonstrates that established IOA is a similarly effective alternative to peripheral IV access.

Regarding the epidemiology of IOA use, non-traumatic CA is the most common situation in which it is used, followed by its use in trauma patients (such as those involved in traffic accidents, falls from height, and burns), cases of shock, respiratory distress, and situations where obtaining peripheral venous access may result in significant time loss or be simply impossible. Therefore, the epidemiological profile aligns with the results obtained from our study (10, 20–23).

## 5 Limitations

The small population of the Autonomous Community of La Rioja could be considered a limitation for the study; despite this, as reflected in the statistics on population, resources, and activity published by the Ministry of Health, La Rioja is the 7th province with the highest number of health care demands per 1,000 inhabitants (227, a total of 71,527 demands during 2022), only behind Gerona, Lerida, Asturias, Navarra, Tarragona, and Barcelona, placing the average for the National Health System at 192. Despite this, La Rioja, in terms of mobilization of class C vehicles (ALSU) for every 1,000 demands for care, is in eighth position, with 61 mobilizations, while the average for the National Health System is 146 mobilizations for every 1,000 inhabitants.

The critical situation of patients requiring IO vascular access, *a priori* a limitation for the collection of data, did not limit the study, and in the end, the involvement of all the healthcare personnel of the Emergency Health Service 061 La Rioja was very high.

## 6 Conclusion

After carrying out this study, we can conclude that the use of IO vascular access in critically ill patients benefits the patient, providing the nursing professional with quick, simple, and uncomplicated access for its installation and use in situations in which cannulation of the peripheral venous line is difficult or impossible, and that the SES of La Rioja used this technique correctly and effectively during the year 2022.

The main use during the year 2022 was in patients in *CA.* It is noteworthy that out-of-hospital stabilization was achieved in all individuals to whom IO vascular access was cannulated and presented a critical situation, shock, and severe trauma.

Professionals with great experience and expertise in the cannulation of peripheral venous lines do not need to make any unsuccessful cannulation attempt to conclude the need for the use of IO vascular access, the key to the outcome of the condition is the professional’s decision-making capacity in this regard, which has a direct impact on the decision taken at the right time, thus avoiding the unfortunate progression of the clinical condition.

IO vascular access provides an adequate route for the administration of drugs and fluids, facilitating out-of-hospital stabilization of the patient and allowing transfer to a useful center when cannulation of a peripheral venous line is not possible.

## Data availability statement

The raw data supporting the conclusions of this article will be made available by the authors, without undue reservation.

## Ethics statement

The studies involving humans were approved by University of La Rioja Ethics Committee. The studies were conducted in accordance with the local legislation and institutional requirements. Written informed consent for participation in this study was provided by the participants’ legal guardians/next of kin.

## Author contributions

AB-E: Conceptualization, Data curation, Formal analysis, Funding acquisition, Investigation, Methodology, Project administration, Resources, Software, Supervision, Validation, Visualization, Writing – original draft, Writing – review & editing. MQ-D: Formal analysis, Funding acquisition, Methodology, Project administration, Supervision, Validation, Writing – original draft, Writing – review & editing, Conceptualization, Data curation, Investigation, Resources, Software, Visualization. VC-H: Investigation, Writing – original draft. MG-L: Investigation, Writing – original draft. IS-A: Conceptualization, Data curation, Funding acquisition, Investigation, Methodology, Project administration, Resources, Writing – original draft. RV-H: Formal analysis, Funding acquisition, Investigation, Project administration, Resources, Visualization, Writing – original draft. VG-C: Conceptualization, Investigation, Methodology, Project administration, Visualization, Writing – original draft. JS-S: Conceptualization, Formal analysis, Methodology, Resources, Writing – original draft. NN-E: Formal analysis, Methodology, Project administration, Writing – original draft. AR-C: Investigation, Project administration, Resources, Writing – original draft. PS-C: Software, Visualization, Writing – original draft. RJ-V: Conceptualization, Data curation, Formal analysis, Funding acquisition, Investigation, Methodology, Project administration, Resources, Software, Supervision, Validation, Visualization, Writing – original draft, Writing – review & editing.

## Footnotes

^1^verification URL: https://sede.unirioja.es/csv/code/nOjsSvXLLNRvnfVfPr299EBR2092SU2L

## Appendix

Appendix I Age and sex of patients.

**Table:**

	Sex and age															
	Male								Female							
			Cardiorespiratory arrest		Shock		Severe trauma				Cardiorespiratory arrest		Shock		Severe trauma	
Age	*N*	%	*N*	%	*N*	%	*N*	%	*N*	%	*N*	%	*N*	%	*N*	%
0–10	2	10.0	2	100	0	0.0	0	0.0	2	22.2	2	100	0	0.0	0	0.0
11–20	0	0.0	0	0.0	0	0.0	0	0.0	0	0.0	0	0.0	0	0.0	0	0.0
21–30	0	0.0	0	0.0	0	0.0	0	0.0	0	0.0	0	0.0	0	0.0	0	0.0
31–40	2	10.0	1	50.0	1	50.0	0	0.0	1	11.1	1	100	0	0.0	0	0.0
41–50	2	10.0	1	50.0	0	0.0	1	50.0	1	11.1	1	100	0	0.0	0	0.0
51–60	5	25.0	3	60.0	1	20.0	1	20.0	2	22.2	2	100	0	0.0	0	0.0
61–70	4	20.0	3	75.0	1	25.0	0	0.0	0	0.0	0	0.0	0	0.0	0	0.0
71–80	4	20.0	4	100	0	0.0	0	0.0	2	22.2	2	100	0	0.0	0	0.0
81–90	1	5.0	1	100	0	0.0	0	0.0	1	11.1	0	0.0	1	100	0	0.0
TOTAL	20	100	15		3		2		9		8		1		0	

Appendix II List of drugs and fluids administered.

**Table:**

Drug	Clinical situation						
	Cardiorespiratory arrest		Shock		Severe trauma		Test de Fisher
	*N*	%	*N*	%	*N*	%	
Adrenaline	16	69.6	0	0	1	50.0	*p*-value = 0.020
Tranexamic acid	0	0	1	25	0	0	*p*-value = 0.207
Amiodarone	2	8.7	0	0	0	0	*p*-value = 1
Atropine	1	4.3	0	0	0	0	*p*-value = 1
Bicarbonate	1	4.3	0	0	0	0	*p*-value = 1
Ketamine	0	0	0	0	1	50.0	*p*-value = 0.068
Labetalol	0	0	1	25.0	0	0	*p*-value = 0.207
Midazolam	3	13.0	1	25.0	1	50.0	*p*-value = 0.269
Ondansetron	0	0	1	25.0	0	0	*p*-value = 0.207
Rocuronium	3	13.0	1	25.0	0	0	*p*-value = 0.627
Somatostatin	0	0	1	25.0	0	0	*p*-value = 0.207
Succinylcholine	2	8.7	1	25.0	0	0	*p*-value = 0.515
Fentanyl	2	8.7	1	25.0	1	50.0	*p*-value = 0.180
Total drug use	17	73.9	2	50.0	2	100.0	*p*-value = 0.583
Fluid infusion							
Yes	17	73.9	4	100	2	100	*p*-value = 0.7167
Not	6	26.1	0	0.0	0	0	
